# Increasing prevalence of ciprofloxacin-resistant food-borne *Salmonella* strains harboring multiple PMQR elements but not target gene mutations

**DOI:** 10.1038/srep14754

**Published:** 2015-10-05

**Authors:** Dachuan Lin, Kaichao Chen, Edward Wai-Chi Chan, Sheng Chen

**Affiliations:** 1 Food Safety and Technology Research Center, Hong Kong PolyU Shenzhen Research Institute, Shenzhen, P. R. China; 2 State Key Lab of Chirosciences, Department of Applied Biology and Chemical Technology, The Hong Kong Polytechnic University, Hung Hom, Kowloon, Hong Kong SAR

## Abstract

Fluoroquinolone resistance in *Salmonella* has become increasingly prevalent in recent years. To probe the molecular basis of this phenomenon, the genetic and phenotypic features of fluoroquinolone resistant *Salmonella* strains isolated from food samples were characterized. Among the 82 *Salmonella* strains tested, resistance rate of the three front line antibiotics of ceftriaxone, ciprofloxacin and azithromycin was 10%, 39% and 25% respectively, which is significantly higher than that reported in other countries. Ciprofloxacin resistant strains typically exhibited cross-resistance to multiple antibiotics including ceftriaxone, primarily due to the presence of multiple PMQR genes and the *bla*_CTX-M-65_, *bla*_CTX-M-55_ *bla*_CMY-2_ and *bla*_*CMY-72*_ elements. The prevalence rate of the *oqxAB* and *aac(6’)-Ib-cr* genes were 91% and 75% respectively, followed by *qnrS* (66%), *qnrB* (16%) and *qnrD* (3%). The most common PMQR combination observable was *aac(6’)-Ib-cr*-*oqxAB*-*qnrS*2, which accounted for 50% of the ciprofloxacin resistant strains. Interestingly, such isolates contained either no target mutations or only a single *gyrA* mutation. Conjugation and hybridization experiments suggested that most PMQR genes were located either in the chromosome or a non-transferrable plasmid. To summarize, findings in this work suggested that PMQRs greatly facilitate development of fluoroquinolone resistance in *Salmonella* by abolishing the requirement of target gene mutations.

Foodborne salmonellosis is one of the leading causes of foodborne illnesses worldwide. Although antimicrobial treatment is usually not necessary due to the self-limiting nature of salmonellosis, it can be lifesaving in cases of invasive infections^1^, with ceftriaxone and ciprofloxacin being the key drugs of choice^2^. Resistance to ceftriaxone or other extended spectrum beta-lactams is usually due to intracellular production of extended spectrum β-lactamases (ESBLs) such as the CTX-M group and AmpC β-lactamase, including the CMY-2 enzyme, which are usually located on transmissible plasmids that tend to disseminate among members of *Enterobacteriaceae*^3^,^4^. Prevalence of resistance to ceftriaxone in *Salmonella* appears to be slowly increasing, reaching a rate of around 3 ~ 4% at present^5^. However, the rate of resistance to ciprofloxacin has increased dramatically both in clinical and food isolates around the world, in particular China and the adjacent areas^6^. Ciprofloxacin resistance is mainly attributed to double mutations in the *gyrA* gene and single mutation in the *parC* gene in *Salmonella*^7^,^8^. Efflux pumps and the presence of plasmid-mediated quinolone resistance (PMQR) determinants have also been regarded as contributive factors of development of low level resistance to nalidixic acid. At least three types of PMQRs have been reported so far including (i) the Qnr types, which are pentapeptide repeat proteins that bind to DNA gyrase by mimicking double stranded DNA, preventing fluroquinolone binding to gyrase, (ii) Aac(6′)-Ib-cr, a modified aminoglycoside acetyltransferase that hydrolyzes fluoroquinolones and (iii) the efflux pumps QeqA, and OqxAB. Unlike *E. coli* and various other members of *Enterobacteriaceae*, the development of mutations in the *gyrA* and *parC* genes in *Salmonella* is known to be a very slow event, resulting in an unusually low level of ciprofloxacin resistance in *Salmonella*. On the other hand, although PMQRs were commonly detectable in *Enterobacteriaceae*, in particular *E. coli*, prevalence of PMQRs in *Salmonella* remains extremely low. To date, a few types of PMQRs including *qnrA*, *qnrB, qnrD,* and *qnrS* alleles have been reported in a limited number of studies^9^,^10^,^11^,^12^,^13^,^14^. Recently, a new PMQR gene, *oqxAB*, which was originally identified on a plasmid (pOLA52) recoverable from *E. coli*, was first reported in *Salmonella* isolates of food origin. The mobile efflux pump OqxAB belongs to the RND-family and shares up to 40% homology with other RND- type efflux systems such as AcrAB in *E. coli* and MexAB in *Pseudomonas aeruginosa*^15^. Exhibiting the ability to enhance the MICs of olaquindox, ampicillin, quinolones and chloramphenicol^6^,^16^, this element was subsequently found to be increasingly prevalent among *Salmonella* isolates recoverable from different sources after the year 2006^6^,^17^. The *oqxAB* operon was suggested to play a functional role which helps accelerate the development of ciprofloxacin resistance in *Salmonella*, and was hence considered to be responsible for causing the recent dramatic increase of ciprofloxacin resistance in clinical *Salmonella* strains^18^.

The combination of PMQR such as *oqxAB* and a single target gene mutation, in particular in the *gyrA* gene, could possibly mediate development resistance to ciprofloxacin in *Salmonella*, and dramatically reduced the time required for the development of a resistance phenotype associated with generation of double *gyrA* mutations and single *parC* mutation^18^. This idea is supported by the observation of an increasing prevalence of different PMQR genes in various species of *Enterobacteriaceae*, and the emergence of ciprofloxacin-resistant *E. coli* and *Salmonella* strains carrying multiple PMQRs without target mutations^19^,^20^. In this study, we reported the high prevalence of ciprofloxacin resistant *Salmonella* strains in food samples, most of which were found to harbor either only a single mutation in *gyrA*, or no mutation in both target genes. However, the strains commonly contained multiple PMQR genes, with the most prevalent being the *oqxAB* and *aac(6′)-Ib-cr* elements. Discovery of this novel phenomenon, in which one bacterial resistance mechanism promotes the onset of another, signals a risk of aggravation of the clinical problem of ciprofloxacin resistance in *Salmonella*. The current situation warrants a need for continuous surveillance of the prevalent mechanisms of ciprofloxacin resistance in *Salmonella* in order to better understand the genetic background of this new category of resistant organisms.

## Results

### High prevalence of antimicrobial resistance in *Salmonella* food isolates

A total of 82 *Salmonella* strains were isolated from chicken and pork samples purchased from supermarkets and wet-markets in Shenzhen, China during the period of November 2012 to June 2013. These *Salmonella* strains were subjected to further characterization of their antimicrobial resistance to various antibiotics (Table 1), and the underlying resistance mechanisms. Overall, these strains exhibited a very high rate of resistance to most of the antibiotics tested. The resistance rate of the three most important front line antibiotics (ceftriaxone, ciprofloxacin and azithromycin) were respectively 10%, 39% and 25%, which is significantly higher than that reported in other countries*. Salmonella* strains isolated from pork samples exhibited a higher rate of resistance to most of the antibiotics tested when compared to *Salmonella* chicken isolates, in particular ciprofloxacin (Table 1). Surprisingly, chicken *Salmonella* isolates exhibited a much higher rate of resistance to ceftriaxone (35%) than that of the pork isolates (11%). This phenomenon is probably due to the high rate of resistance to ceftriaxone in *S.* Indiana. Among the different serotypes tested, *S.* Indiana also exhibited the highest rate of resistance to most antibiotics, including the three front line drugs of ceftriaxone, ciprofloxacin and azithromycin. Such phenotype has only been reported in *S.* Typhimurium and *S.* Kentucky previously^6^,^21^. *S.* Typhimurium and *S.* Derby also exhibited a very high resistance rate except that *S.* Typhimurium did not exhibit resistance to ceftriaxone. Two serotypes, namely *S.* Heidelberg and *S.* Rosentha, exhibited an intermediate rate of resistance to the test antibiotics. On the other hand, resistance was less commonly observed among *S.* Enteritidis. Another important observation is that, among 32 ciprofloxacin-resistant strains, all were resistant to ampicillin, nalidixic acid, kanamycin, streptomycin, chloramphenicol, tetracycline and sulfamethoxazole; furthermore, the MIC of olaquindox was generally 32 mg/L or higher for these strains. We also observed that, among such isolates, up to 84% were resistant to gentamicin, 25% were resistant to azithromycin (MIC ≥ 32 mg/L), and 13% were also resistant to ceftriaxone (Table 2).

### Diverse mechanisms of ceftriaxone resistance in *Salmonella* food isolates

Mechanisms of resistance in selected strains, in particular those mediating resistance to the front line antibiotics such as ceftriaxone and ciprofloxacin, were investigated. Resistance to ceftriaxone was detectable in 8 *Salmonella* isolates including 4 *S.* Indiana, 2 *S.* Heidelberg, 1 *S.* Enteritidis and 1 *S.* Derby. These 8 strains were examined for their ability to produce Extended-spectrum β-lactamases and AmpC β-lactamases, with results showing that diverse resistance mechanisms were observable. Three out of the four *S.* Indiana strains were found to contain *bla*_CTX-M-65_, with the fourth one harboring the *bla*_*CMY-2*_ gene. The *bla*_CTX-M-55_ gene was detectable in one *S.* Enteritidis and one *S.* Derby strain. For the two *S.* Heidelberg isolates, the *bla*_CMY-2_ and bla_CMY-72_ genes were each detectable in one strain (Table 2).

### Novel mechanisms of fluoroquinolone resistance in *Salmonella* food isolates

A total of 32 ciprofloxacin-resistant *Salmonella* strains were subjected to investigation of the mechanisms involved. Contrary to the resistance mechanisms commonly observable in clinical ciprofloxacin resistant strains, in which double and single mutations often occur in the *gyrA* and *parC* genes respectively, most of the 32 ciprofloxacin resistant *Salmonella* strains tested in this work were found to contain either only a single mutation in *gyrA*, with S83T, S83F, and D87N being the most common amino acid changes, or no mutation in both target genes (Table 2). The few exceptions were all *S.* Indiana isolates which harbored the double *gyrA* mutations S83F and D87N, with or without the single *parC* mutation S80R. It should also be noted that a pair of novel double *gyrA* mutations which resulted in the H80N and S83T changes, and single *parC* mutation causing the Q91H substitution, were detectable in a *S.* Rissen isolate; however, the roles of such mutations in development of *Salmonella* fluoroquinolone resistance are not well defined at present. Other less common mutations that were detectable include the C72G change in the *parC* gene product of a *S.* Indiana strain, and a S83I change in the GyrA protein of a *S.* Derby isolate. The nature of contribution of these novel mutations to the development of ciprofloxacin resistance in *Salmonella* needs further investigation. No mutations were detected in *gyrB* and *parE*.

The presence of PMQR genes in ciprofloxacin resistant *Salmonella* isolates were also screened by PCR and sequencing (Table 2). Surprisingly, all isolates were found to carry PMQRs, with *oqxAB* and *aac(6′)-Ib-cr*, the most prevalent elements, reaching a rate of 91% and 75% respectively. Other PMQR genes detectable included *qnrS* (66%), *qnrB* (16%) and *qnrD* (3%). The most common PMQR combination observable was *aac(6′)-Ib-cr*-*oqxAB*-*qnrS*2, which accounted for 50% of all the ciprofloxacin resistant *Salmonella* strains tested. To determine if other resistance mechanisms such as efflux activities contribute to ciprofloxacin resistance in such isolates, the MIC of ciprofloxacin against these isolates was determined in presence and absence of the efflux pump inhibitor, Phenylalanine-arginine β-naphthylamide (PAβN). The results showed that PAβN caused a mild reduction in the MIC level, suggesting that drug efflux only played a partial role in ciprofloxacin resistance development in these organisms (Table 2). Detailed analysis of the relative roles of PMQRs and target gene mutations in conferment of ciprofloxacin resistance phenotypes suggested that several PMQRs including *aac(6′)-Ib-cr*, *oqxAB* and *qnrS*, alone or in combination, could mediate ciprofloxacin resistance development in *Salmonella* isolates which did not contain target gene mutations. In particular, the presence of four different PMQRs, such as the *aac(6′)-Ib-cr*-*oqxAB*-*qnrS*2-*qnrB*8 and aac-*oqxAB*-*qnrS*2-*qnrD* combinations, was consistently observable in ciprofloxacin-resistant *Salmonella* isolates without any target mutations, suggesting that effects of such elements in conferring antibiotic resistance in *Salmonella* are additive or synergistic in nature (Table 2). It should also be noted that PMQR-mediated ciprofloxacin resistance is commonly associated with a MIC level of 4 to 8 μg/ml, which is comparable to those conferred by target mutations.

**Figure 1:**
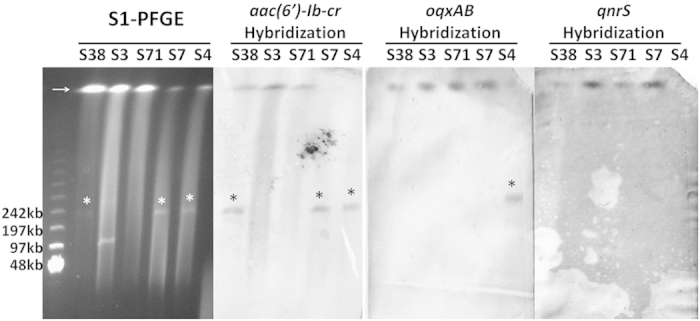
Analysis of genetic location of specific PMQRs in specific ciprofloxacin resistant strains by S1-PFGE and Southern hybridization. The arrow denotes the position of chromosomal DNA. An asterisk denotes plasmid band in which positive hybridization signal was detectable.

Conjugation experiments were performed on these 32 ciprofloxacin-resistant *Salmonella* isolates to confirm if PMQRs were readily transferable to other *Enterobacteriaceae* species, using the *E. coli* J53 strain as recipient. Surprisingly, none of the ciprofloxacin resistance phenotypes tested could be transferred to *E. coli*, suggesting these PMQR genes may be present on a non-conjugative plasmid or the chromosomal DNA of *Salmonella*. To test these possibilities, five representative *Salmonella* strains including *S.* Derby strains S3, S4 and S38, and *S.* Typhimurium strains S7 and S71, all exhibiting different PFGE types and harboring different PMQRs, were selected for S1-PFGE and southern hybridization analysis (Fig. 1). Among these isolates, the *aac(6′)-Ib-cr* gene was shown to be located in both chromosomal DNA and a ~200 kb size plasmid of the *S.* Derby strain S38, in the chromosome of the *S.* Derby strain S3 and *S.* Typhimurium strain S71, and in a ~200 kb plasmid in the *S.* Derby strain S4 and *S.* Typhimurium strain S7 (Fig. 1). The *oqxAB* gene was found to be located in the chromosome of all the three *S.* Derby strains and the *S.* Typhimurium strain S71, as well as in the same ~200 kb plasmid of *S.* Typhimurium strain S7 which also harbored the *aac(6′)-Ib-cr* gene as aforementioned. The *qnrS* element was shown to be located in the chromosomal DNA of all *S.* Derby strains and the *S.* Typhimurium strain S71, but not in strain S7. Such findings are consistent with the PCR screening results (Fig. 1, Table 2). Hybridization was also performed to probe the location of *qnrB* that was present in *S.* Typhimurium strain S71. However the hybridization experiment was not successful even though the presence of *qnrB* in this strain has been confirmed by both PCR and sequencing.

**Figure 2:**
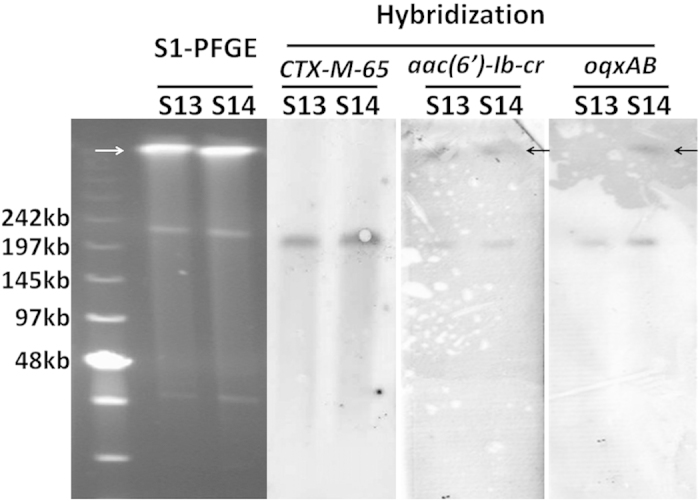
Analysis of genetic location of the *bla*_CTX-M-65_ element and specific PMQRs in specific ciprofloxacin and ceftriaxone resistant strains by S1-PFGE and Southern hybridization. The arrow denotes the position of chromosomal DNA. An asterisk denotes plasmid band in which positive hybridization signal was detectable.

*Salmonella* isolates that exhibited resistance to both ceftriaxone and ciprofloxacin included three *S.* Indiana strains, S13, S14 and S16. Again, conjugation experiments failed to transfer either the ciprofloxacin or ceftriaxone resistance phenotype to *E. coli* J53. Southern hybridization was performed on strains S13 and S14 to determine the genetic location of the *bla*_CTX-M-65_ element and the PMQR genes. Our data demonstrated that the *bla*_CTX-M-65_, *oqxAB* and *aac(6′)-Ib-cr* elements were all located on the same ~200 kb plasmid (Fig. 2). However, hybridization experiment performed to confirm the genetic location of the *qnrB* gene in strain S14 was not successful even though it was proven to be present in the isolate by PCR.

## Discussion

PMQRs play an important role in the development of fluoroquinolone resistance in *Enterobacteriaceae*^18^,^19^. These elements have mainly been reported in *E. coli* strains isolated from various sources and their prevalence has been shown to increase dramatically in recent years. In contrast, PMRQs have only been recoverable from *Salmonella* since 2005; nevertheless, their prevalence remains extremely low in *Salmonella* until the emergence of a new PMQR determinant, namely *oqxAB*, which encoded an efflux pump mediating resistance to olaquindox, chloramphenicol, nalidixic acid and elevated MICs of other antimicrobial reagents including ampicillin and gentamicin^16^. The *oqxAB* operon was first found to be present in an IncX1 type plasmid designated as pOLA52, which was recoverable from swine *Escherichia coli* isolates^15^,^22^. More recently, *oqxAB* was reported to be prevalent in organisms isolated from pork as well as pig farms in China^23^,^24^,^25^. In fact, various lines of evidence suggest that this mobile resistance element already existed in poultry *E. coli* isolates as early as 1994^23^. On the other hand, *oqxAB* has not been found in clinical isolates until recently, when it became detectable in clinical strains of *E. coli* and *Klebsiella pneumoniae*^26^,^27^,^28^,^29^.

In *Salmonella*, *oqxAB* was first found to be present in the chromosomal DNA of two *S.* Derby strains of food origin in 2013^17^. Retrospective study of clinical isolates of *Salmonella* in China revealed that *oqxAB* could be detected in *Salmonella* as early as 2006^6^, and that it was often genetically associated with the *aac(6′)-Ic-br* element, contributing to transmission of drug resistant organisms in clinical setting in both clonal and non-clonal manner. Further studies showed that *oqxAB* and *aac(6′)-Ic-br* could greatly facilitate development of fluoroquinolone resistance by abolishing the requirement of target gene mutations, thereby potentially causing a dramatic increase of fluoroquinolone resistance in *Salmonella*^18^. Our data confirmed that this is indeed the case, and suggested that by further acquiring other PMQRs in *Salmonella* strains which already harbored the *oqxAB* and *aac(6′)-Ib-cr* elements, fluoroquinolone resistance at a level comparable to that conferred by target mutations is consistently achievable in organisms that do not even harbor a single *gyrA* mutation. The finding that multiple PMQR elements can simultaneously or synergistically produce a fluoroquinolone resistance phenotype via the mechanisms of enzymatic inactivation, drug efflux, and competitive inhibition of drug binding is intriguing. Since *oqxAB* or other PMQR determinants that confer reduced susceptibility of the host organism to fluoroquinolones may also enhance the rate of mutational changes in the drug target gene (12), the phenomenon of rapid transmission of PMQR elements among members of *Enterobacteriaceae* is alarming.

Although our data showed that the PMQRs detectable in *Salmonella* were often found to be located in chromosome or plasmids that could not be transferred to other bacterial through conjugation, these elements must have been, at some stages, harbored by mobile elements that are capable of transferring its contents to the chromosome of the host strain via transposition events. This was evidenced by the observation that the *oqxAB* element can be recoverable in both chromosome and mobile elements containing the IS*26* element^17^,^18^. With the fast progress of *oqxAB*-associated PMQR evolution in *Salmonella*, transmission of plasmids mediating fluoroquinolone resistance among *Salmonella*, or between *Salmonella* and other bacterial species, may become even more efficient, posing a huge threat to *Salmonella* infection control in clinical settings. Plasmids carrying the *bla*_CTX-M-65_ gene and multiple PMQR cassettes are of particular concern, despite the fact that they are currently restricted to specific strains such as *S.* Indiana.

Olaquindox has been a widely used growth enhancer in the pig-raising industry since the 1970s^30^,^31^. Its antibiotic activity can be attributed to its ability to inhibit DNA synthesis. This agent was previously considered safe since they were not structurally related to any human drug. Findings in this work constitute part of the evidence that the use of olaquindox as growth promoter in the swine industry has resulted in some unexpected consequences, the impact of which only became evident decades later. First, our recent study confirmed that *oqxAB* actually originated as a chromosomal efflux pump gene of *Klebsiella pneumoniae*, which was picked up and incorporated into a mobile element by IS*26*-mediated transposition, presumably under the selection pressure of olaquindox. These events resulted in constitutive expression of the plasmid-borne *oqxAB* operon. Second, the process of inter-species transmission from *E. coli* to *Salmonella* occurred over a period of at least a decade, during which *oqxAB* was not detectable in clinical *Salmonella* strains until 2006. Third, amplification of an *oqxA* –positive *S.* Typhimurium strain resulted in a sharp increase in the prevalence of *oqxAB*-borne clinical *Salmonella* isolates in subsequent years. Finally, our data demonstrated that co-existence of the *oqxAB* genes with other PMQR elements has become commonplace, leading to emergence of a new category of fluoroquinolone-resistant organisms that exhibit selective advantages in both the environment and clinical settings where antibiotic selection pressure is high. At present, *Salmonella* strains harboring multiple PMQR/*oqxAB* elements appear to be confined to zoonotic organisms but the risk of these strains causing human infections is apparently increasing rapidly. To conclude, findings in this work highlight a need to devise specific infection control measures to halt further transmission of the *oqxAB*/PMQR–borne resistant *Salmonella* strains, and investigate the impact of other animal growth promoters in selection of both bacterial resistance and virulence determinants in a wide range of foodborne and zoonotic pathogens.

## Materials and Methods

### *Salmonella* isolation from retail meat products

*Salmonella* were isolated from retail meat samples including chicken and pork from supermarkets and wet markets in Shenzhen, China from October 2012 to June 2013^32^. Food samples were collected aseptically in plastic bags and transported on ice to the laboratory for isolation of *Salmonella* within 6 h. Twenty-five grams of meat samples were placed in a stomacher bag with 100-ml Buffered Peptone Water (BPW) (Difco, Detroit, MI) which was subjected to homogenization for 5 min. The homogenate was incubated at 35 °C for 24 h. One ml aliquot of pre-enriched homogenate was transferred to 10 mL of Tetrathionate broth (Difco) and incubated at 42 °C for 24-h. A loopful of the enriched content was streaked on XLT4 agar and incubated for 24 h to 48 h at 37 °C. One typical *Salmonella* strain recovered from each sample was purified and subjected to species identification by detection of the *invA* gene and 16S RNA sequencing. All isolates were serotyped according to the Kauffmann-White scheme, using commercial antiserum (Difco, Detroit).

### Antimicrobial susceptibility tests

Confirmed *S.* Typhimurium isolates were subjected to antimicrobial susceptibility testing using the agar-dilution method, and the results were interpreted according to the CLSI guidelines^33^. Fourteen antimicrobial agents were tested: ampicillin, cefotaxime, ceftriaxone, amoxicillin/clavulanic acid, sulfamethoxazole, kanamycin, amikacin, gentamicin, tetracycline, chloramphenicol, ciprofloxacin, nalidixic acid, streptomycin, and olaquindox. *E.coli* strains ATCC 25922 and 35218, *Enterococcus faecalis* strain ATCC 29212, *Staphylococcus aureus* strain ATCC 29213 and *Pseudomonas aeruginosa* ATCC 27853 were used as quality control.

### Screening of target gene mutations, *oqxAB*, and other PMQR genes

The QRDR regions of the *gyrA, gyrB, parC* and *parE* genes were amplified by PCR as previously described^34^, followed by determination of their nucleotide sequences and comparison to the wild-type *Salmonella* Typhimurium LT2 strain for identification of target gene mutations in the test strains. The presence of the PMQR genes *qnrA, qnrB, qnrC, qnrD, qnrS, qepA, oqxAB* and *aac(6′)Ib-cr*, was determined by PCR using primers described previously^34^,^35^.

### Molecular typing

Clonal relationship between representative *Salmonella* isolates was examined by pulsed-field gel electrophoresis (PFGE) according to the PulseNet PFGE protocol for *Salmonella*^36^. S1-PFGE was conducted to determine the size of large plasmids. Briefly, agarose-embedded DNA was digested with S1 nuclease (New England Bio-Lab) at 37 °C for 1 hr. The restriction fragments were separated by electrophoresis in 0.5 Tris-borate-EDTA buffer at 14 °C for 18 h using a Chef Mapper electrophoresis system (Bio-Rad, Hercules, CA) with pulse times of 2.16 to 63.8 *S.* Phage Lambda PFGE ladder (New England Biolab) was used as DNA size marker. The gels were stained with GelRed, and DNA bands were visualized with UV transillumination (Bio-Rad). Southern blot hybridization was carried out by following the manufacturer’s instructions of the DIG-High Prime DNA Labeling and Detection Starter Kit II (Roche Diagnostics), using the different PMQR gene and *bla*_CTX-M-64_ digoxigenin-labeled probes.

## Additional Information

**How to cite this article**: Lin, D. *et al.* Increasing prevalence of ciprofloxacin-resistant food-borne *Salmonella* strains harboring multiple PMQR elements but not target gene mutations. *Sci. Rep.* **5**, 14754; doi: 10.1038/srep14754 (2015).

## Figures and Tables

**Table 1:** Prevalence of antimicrobial resistance in different *Salmonella* serotypes.

Antimicrobials	% of Resistance								
	Overall (n = 82)	Chicken isolates (n = 29)	Pork isolates (n = 53)	*S.* Derby (n = 32)	*S.* Typhimurium (n = 16)	*S.* Heidelberg (n = 8)	*S.* Rosentha (n = 8)	*S.* Indiana (n = 4)	*S.* Enteritidis (n = 4)
Ampicillin	68	62	72	69	100	100	63	100	25
Cefotaxime	10	21	4	3	0	25	0	100	25
Ceftriaxone	10	21	4	3	0	25	0	100	25
Chloramphenicol	74	69	75	83	79	100	50	75	50
Gentamicin	40	19	47	52	43	25	0	100	0
Kanamycin	48	27	55	69	64	25	25	100	0
Streptomycin	50	42	53	59	57	63	25	100	0
Nalidixic acid	63	46	68	72	57	50	38	100	25
Ciprofloxacin	39	17	51	50	57	0	0	75	0
Sulfamethoxazole	100	100	100	100	100	100	100	100	100
Tetracycline	65	42	75	76	79	63	63	100	0
Amikacin	4	4	4	0	0	0	0	75	0
Azithromycin	25	23	31	38	6	13	25	75	0
Olaquidox	51	35	58	90	71	50	25	75	25

**Table 2:** Phenotypic and genotypic characteristics of *Salmonella* strains isolated from retail meat products.

Strain #	Isolation date	Sources	Serotypes	PFGE	Resistance Profiles*	CIP	CIP/PAβN	PMQRs	Mutations in *gyrA*	Mutations in *parC*
S3^a^	12/12/12	P	Derby	DER1	Gen	16	2	aac-*oqxAB*-*qnrS*2	—	—
S24	01/12/13	P	Derby	DER3	Gen	4	2	*oqxAB*	S83I	
S35	01/26/13	P	Derby	DER5	Gen	4	4	aac-*oqxAB*-*qnrS*2	S83T	—
S36	01/26/13	P	Derby	DER5	Gen-Azi	8	2	aac-*oqxAB*-*qnrS*2	—	—
S37	01/26/13	P	Derby	DER5	Gen	4	2	aac-*oqxAB*-*qnrS*2	—	—
S38^a^	01/26/13	P	Derby	DER5	Gen-Azi	2	2	aac-*oqxAB*-*qnrS*2	N78H	—
S39	01/26/13	P	Derby	DER5	Gen	8	4	aac-*oqxAB*-*qnrS*2	—	—
S40	01/26/13	P	Derby	DER5	Gen-Azi	8	4	aac-*oqxAB*-*qnrS*2	—	—
S41	01/26/13	P	Derby	DER5	Gen-Azi	4	4	aac-*oqxAB*-*qnrS*2	—	—
S42	01/26/13	P	Derby	DER5	Gen	4	2	aac-*oqxAB*-*qnrS*2	—	—
S44	01/26/13	P	Derby	DER5	Gen	4	4	aac-*oqxAB*-*qnrS*2	—	—
S4^a^	12/12/12	P	Derby	DER6	Gen	2	2	aac-*oqxAB*-*qnrS*2-*qnrB*8	—	—
S9	12/12/12	P	Derby	DER6		4	0.5	aac-*oqxAB*-*qnrS*2	S83T	—
S54	03/13/13	C	Derby	DER8		2	0.12	*oqxAB*-*qnrS*8-*qnrB*	S83T	
S49	03/03/13	P	Derby	DER10	Gen-Azi	>32	>8	*oqxAB*-*qnrS*1-*qnrB*	S83T	
S48	03/03/13	C	Derby	DER11	Gen	2	0.5	*qnrS*1	S83T	—
S7^a^	12/12/12	P	Typhimurium	TR1	Gen	4	0.25	aac-*oqxAB*	D87N	—
S8	12/12/12	P	Typhimurium	TR1		4	0.25	*oqxAB*	D87N	—
S11	12/12/12	P	Typhimurium	TR1	Gen	2	0.25	aac-*oqxAB*	D87N	—
S79	05/17/13	P	Typhimurium	TR2	Gen	4	0.12	*oqxAB*	S83F	—
S65	03/21/13	P	Typhimurium RH2	TRH1	Gen	2	0.5	aac-*oqxAB*-*qnrS*1	D87N	—
S66	03/21/13	P	Typhimurium RH2	TRH2	Gen	4	0.5	aac-*oqxAB*-*qnrS*1	—	—
S71^a^	05/01/13	P	Typhimurium RH2	TRH2	Gen	>32	>8	aac-*oqxAB*-*qnrS*1	D87N	—
S6	12/12/12	P	Typhimurium RH2	TRH2	Gen	4	0.25	aac-*oqxAB*-*qnrS*1	—	—
S20	01/12/13	C	Typhimurium RH2	TRH3	Gen	2	0.5	aac-*qnrB*	D87N	—
S13	12/25/12	P	Indiana	I1	Gen-Azi-Cro	>32	>8	aac-*oqxAB*	S83F, D87N	—
S14	12/25/12	C	Indiana	I1	Gen-Azi-Cro	>32	>8	aac-*oqxAB*-*qnrB*	S83F, D87N	S80 R
S16	12/25/12	P	Indiana	I1	Gen-Azi-Cro	>32	>8	aac-*oqxAB*	S83F, D87N	C72G, S80 R
S27	01/19/13	P	Rissen	R1		8	0.12	*oqxAB*	H80N, S83T	Q91H
S59	03/16/13	C	London	L1		2	0.12		D87N	
S2	12/12/12	P	Sanferberg	S1	Gen	4	4	aac-*oqxAB*-*qnrS*2	S83T	—
S45	02/22/13	P	Virchow	V2	Gen	4	4	aac-*oqxAB*-*qnrS*8-qnrD	—	—

^*^All isolates were resistant to the antibiotic profile of Amp-Cip-Nal-Kan-Str-Chl-Tet-Sul-Ola (olaquindox); specific strains were also resistant to Gen, gentamicin; Azi, azithromycin; and Cro, ceftriaxone. PaβN, Phenylalanine-arginine β-naphthylamide. C, Chicken; P, Pork; aac, *aac(6′)-Ib-cr*;

^a^selected for S1-PFGE and Southern hybridization.
